# *Porphyromonas gingivalis* promotes T-cell homing via the NF-κB/CXCR3 signaling pathway in experimental autoimmune encephalomyelitis

**DOI:** 10.1080/20002297.2026.2736905

**Published:** 2026-09-26

**Authors:** Dong Dai, Shengyuan Huang, Guoqin Cao, Min Xu, BoYu Cui, Qiuying Ma, Jiang Lin

**Affiliations:** a Department of Stomatology, Beijing Tongren Hospital, Capital Medical University, Beijing, China; b Department of Neurology, Beijing Tongren Hospital, Capital Medical University, Beijing, China

**Keywords:** *Porphyromonas gingivalis*, periodontitis, experimental autoimmune encephalomyelitis, T cells, CXCR3, homing, NF-κB signaling

## Abstract

**Background:**

Multiple sclerosis (MS) is a chronic autoimmune disease of the central nervous system (CNS); experimental autoimmune encephalomyelitis (EAE) is its model. Periodontitis involving *Porphyromonas gingivalis* (*P. gingivalis*) has been linked to MS and shown to exacerbate EAE, but mechanisms remain unclear.

**Methods:**

Mice were randomized into four groups (*n* = 8): control, periodontitis only, EAE only, and EAE with periodontitis. Neurological deficits were assessed daily. Spinal CD4⁺ and CD4⁺CXCR3⁺ T-cell infiltration and NF-κB activation were evaluated by immunofluorescence. Peripheral CD4⁺CXCR3⁺ T-cell frequencies were quantified by flow cytometry. T-cell chemotaxis and CXCR3 expression were assessed.

**Results:**

*P. gingivalis* -induced periodontitis significantly aggravated EAE neurological deficits from days 15 to 21. RNA-seq re-analysis revealed NF-κB signaling enrichment and **Cxcr3*, *Cxcl10*,* and *Nfkb* upregulation. *In vivo, *P. gingivalis** increased peripheral CD4⁺CXCR3⁺ T cells, spinal CD4⁺ and CD4⁺CXCR3⁺ T-cell infiltration, and NF-κB activation. *In vitro, *P. gingivalis** promoted CXCR3-dependent T-cell migration. NF-κB inhibition attenuated *P. gingivalis* -induced NF-κB activation, downregulated CXCR3 expression, and reduced CD4⁺CXCR3⁺ T cells.

**Conclusion:**

*P. gingivalis* -induced periodontitis aggravates EAE clinical severity, potentially involving NF-κB activation, CXCR3 upregulation on peripheral T cells and enhanced T-cell migratory capacity and infiltration into the CNS. These findings identify a potential mechanistic link between periodontitis and MS pathogenesis.

## Key message

- *P. gingivalis*-induced periodontitis is associated with NF-κB activation and higher CXCR3 expression on peripheral CD4⁺ T cells in EAE.
- Elevated spinal cord infiltration of CD4⁺CXCR3⁺ T cells correlates with worsened neurological deficits, suggesting a link between periodontitis and MS-like neuroinflammation.
- Targeting the NF-κB–CXCR3 axis in peripheral T cells may represent a potential adjunctive strategy for managing multiple sclerosis, particularly in patients with comorbid periodontal disease.

## Introduction

Multiple sclerosis (MS), a common autoimmune disease of the central nervous system (CNS), is characterized by inflammation and demyelination and is a leading cause of disability in young adults [1–5]. Experimental autoimmune encephalomyelitis (EAE), which closely resembles MS in clinical and pathological features, is widely used as an ideal animal model for MS research [6–9]. In EAE pathogenesis, Th1 cells dominate the peripheral immune response and can independently induce severe CNS inflammation [10–13]. The principal mechanism involves activated peripheral CD4⁺ T cells—particularly Th1 and Th17 subsets—crossing the blood–brain barrier (BBB) into the CNS, releasing proinflammatory cytokines, and triggering myelin and neuronal injury [1,14,15]. This homing of peripheral T cells is a complex, tightly regulated process orchestrated by chemokines and adhesion molecules. In MS and EAE, the inflammatory CNS milieu induces high expression of chemokines such as CXCL9, CXCL10 and CCL2 [16,17], along with adhesion molecules such as ICAM-1 and VCAM-1 [17]. These ligands bind to corresponding receptors on T cells, including CXCR3, CCR7, CCR2, LFA-1 and VLA-4 [18,19], thereby promoting T-cell migration and homing to sites of inflammation.

Periodontitis is a chronic inflammatory disease caused by periodontal pathogens and is one of the most prevalent chronic diseases worldwide [20–24]. Increasing evidence indicates its association with multiple systemic conditions, including MS [25–31]. *Porphyromonas gingivalis* (*P. gingivalis*), a keystone periodontal pathogen [32–34], is found at elevated abundance in patients with MS and individuals with periodontitis have a higher risk of developing MS [35,36]. Oral administration of *P. gingivalis* has been shown to exacerbate EAE symptoms in mice [37], although the underlying mechanisms remain unclear. Notably, Th1 cells in periodontal lesions exhibit significantly elevated CXCR3 expression [38]. Moreover, CXCR3 antagonists effectively inhibit *P. gingivalis* lipopolysaccharide (LPS)-induced osteoclastogenesis and bone resorption [39,40], underscoring the pivotal role of CXCR3 in periodontal immune responses.

Given that CXCR3–CXCL10 signaling represents an important, though not exclusive, chemokine axis involved in T-cell migration in CNS autoimmunity [41–45], periodontitis may aggravate EAE by upregulating CXCR3 expression on peripheral T cells, thereby enhancing their homing to the CNS and amplifying neuroinflammation. However, whether *P. gingivalis*-induced periodontitis promotes EAE progression through this mechanism has not been clearly elucidated. Therefore, in this study, we aimed to determine whether *P. gingivalis*-induced periodontitis exacerbates EAE progression by enhancing the homing capacity of peripheral T cells through CXCR3 upregulation, and to elucidate the underlying molecular pathways. Our findings may provide new insights into the immunopathological link between periodontitis and MS and help identify potential therapeutic targets for MS.

## Materials and methods

### Animals and experimental groups

Thirty-two female C57BL/6J mice (6–8 weeks; Beijing Vital River Laboratory Animal Technology Co., Ltd.) were housed under specific pathogen-free conditions at the Animal Experimental Center of Beijing Meide Kangna. Female mice were selected to maintain methodological consistency with commonly used MOG_35–55_-induced EAE protocols and comparability across experimental groups. Mice were acclimatized for a minimum of 3 days prior to experimentation. All procedures were approved by the Animal Experimental Ethics Committee of Beijing Meide Kangna and conducted in accordance with Institutional Animal Care and Use Committee guidelines. Animals were randomly assigned (*n* = 8/group) into four groups: healthy control (CON), periodontitis only (*P*), EAE only (EAE) and EAE with periodontitis (EAE + P). The sample size (*n* = 8/group, *n* = number of mice) was chosen based on prior EAE studies with comparable endpoints and variability, while minimizing animal use in accordance with ethical principles. For flow cytometry, immunofluorescence and Western blot analyzes, animals from the same cohort were randomly selected as a subset for tissue collection at the endpoint.

### Culture of *P. gingivalis*

*P. gingivalis* (strain W83, BNCC) was cultured anaerobically on enriched sterile blood agar containing 3.7% brain heart infusion (BHI), 2% agarose, 0.05% L-cysteine, 0.0005% hemin, 0.0001% menadione and 5–7% defibrinated sheep blood. Anaerobic conditions were generated using an AnaeroPack system (MGC, Japan). After 4–5 days of incubation, a single well-isolated colony was aseptically inoculated into BHI broth (3.7% BHI, 0.05% L-cysteine, 0.0005% hemin, 0.0001% menadione) and cultured under anaerobic conditions for an additional 48 h.

### Induction of EAE

EAE was induced by subcutaneous immunization with a 10 mg/mL solution of myelin oligodendrocyte glycoprotein (MOG_35–55_) peptide (QYAOBIO, China) emulsified 1:1 (v/v) with complete Freund's adjuvant (Sigma-Aldrich, USA) containing 5 mg/mL *Mycobacterium tuberculosis* H37Ra [46]. A total of 100 µL of emulsion was administered at four sites along the lower back (25 µL/site) in a single immunization. On Day 0 and again within 48 h, each mouse received an intraperitoneal injection of 200 µL pertussis toxin (GlpBio, China) at 1 µg/mL. Group allocation was performed using a random number table. From the day of immunization, neurological deficits were evaluated daily using Weaver's 15-point neurological function scale [47] (Table 1). Neurological scoring was independently performed by two investigators blinded to group allocation. This scale characterizes disease progression by assessing each limb individually.

**Table 1.** Weaver's 15-point neurological function scale.

Body part	Score	Clinical observation
Tail	0	No symptoms.
	1	Reduced tail tone or partial paralysis.
	2	Full tail paralysis.
Limb (each)	0	No symptoms.
	1	Unstable gait.
	2	Mild paralysis with limb dragging during walking.
	3	Severe paralysis with limb outwardly flipped during walking.

Note: The total score ranges 0–15 and is the sum of the disease status scores for the tail (0–2) and all four limbs (0–3 each). Based on this system, a fully tetraplegic mouse receives a score of 14, and a deceased mouse receives 15.

### Establishment of a periodontitis model

Periodontitis was induced by placing *P. gingivalis*–soaked 6–0 silk ligatures around the maxillary second molars 7 days before EAE induction. Mice then received daily oral inoculation with 100 µL of *P. gingivalis* suspension (1 × 10^9^ CFU/mL) for 28 consecutive days. Successful model establishment was confirmed by consistent clinical signs of gingival inflammation and histological evidence of inflammatory cell infiltration.

### Histopathology

Decalcified tooth–gingiva–alveolar bone blocks were harvested and fixed in 4% paraformaldehyde, followed by decalcification in EDTA until adequately softened. Samples were then rinsed, dehydrated through graded ethanol (70, 80, 95 and 100%), cleared in xylene and embedded in paraffin. Serial sections (3–5 μm) were cut and mounted on glass slides, baked at 60 °C, deparaffinized in xylene and rehydrated through graded ethanol to water. Sections were stained with hematoxylin and eosin (H&E), dehydrated, cleared and coverslipped. Periodontal inflammation and alveolar bone resorption were evaluated histologically.

### Immunofluorescent staining

Paraffin-embedded spinal cord sections were baked at 60 °C for 1 h, deparaffinized in xylene and rehydrated using graded ethanol. Antigen retrieval was performed by boiling in Tris-ethylenediamine tetra-acetic acid buffer (pH 9.0) for 15 min. Sections were blocked with 5% goat serum (Servicebio, China) for 1 h and incubated overnight at 4 °C with rat anti-mouse CD4 (Servicebio, China) and rabbit anti-mouse CXCR3 (Servicebio, China) for CD4/CXCR3 staining or with rat anti-mouse CD4 (Servicebio, China) and rabbit anti-mouse NF-κB p65 (Servicebio, China) for CD4/p65 staining.

After phosphate-buffered saline (PBS) washes, Alexa Fluor 488 goat anti-rat IgG (Servicebio, China) and Alexa Fluor 647 goat anti-rabbit IgG (Servicebio, China) were applied for 1 h in the dark. Nuclei were counterstained with 4',6-diamidino-2-phenylindole (~1 µg/mL; Servicebio, China) and imaged using a Leica TCS SP5 confocal microscope (63×) with optimized laser settings. Image acquisition and quantitative analyzes were performed by investigators blinded to group allocation using predefined criteria.

### Flow cytometry

Flow cytometry was performed to quantify CD4^+^CXCR3^+^ T cells in murine peripheral blood and splenic lymphocytes. Cells were stained for viability using the Zombie NIR™ Fixable Viability Kit (BioLegend, USA) for 15 min in the dark, washed with PBS and then incubated for 20 min in the dark with Brilliant Violet 421™ anti-mouse CD4 (BioLegend, USA) and APC anti-mouse CXCR3 (BioLegend, USA). For in vivo measurements, cells were analyzed directly without in vitro culture; for in vitro assays, splenic lymphocytes were first incubated with *P. gingivalis* for 6 h at 37 °C and then processed using the same staining protocol. Data were acquired on a Cytek NL-CLC3000 flow cytometer (Cytek, USA), with > 10,000 CD4^+^ events collected for each sample. Gating included FSC-A versus FSC-H to exclude doublets, FSC-A versus SSC-A to remove debris and Zombie NIR–negative gates to select live cells. CXCR3 expression was then analyzed within the CD4^+^ T-cell population.

### Reference-based mRNA-seq

Transcriptomic data were obtained from our previously published dataset (NCBI BioProject accession: PRJNA1233427), which includes mRNA-seq of CD4⁺ T cells isolated from the spleens of EAE and EAE + P mice at day 17 post-immunization (*n* = 4 per group).

For the present study, raw sequencing reads were downloaded and reprocessed. Quality control was performed to remove low-quality reads, adapter contamination and poly-*N* sequences. Clean reads were aligned to the *Mus musculus* reference genome (mm10) using the STAR aligner. Polymerase chain reaction (PCR) duplicates were marked using Picard, and single nucleotide polymorphisms were called using the Genome Analysis Toolkit. Gene expression levels were quantified using HTSeq-count. Differential expression analysis was performed using DESeq2 (when biological replicates were available) or DEGseq (when applicable).

Gene ontology (GO) enrichment analysis of differentially expressed genes (DEGs) was conducted using the GOseq R package, and Kyoto Encyclopedia of Genes and Genomes (KEGG) pathway analysis was performed using KOBAS. Protein–protein interaction networks were predicted via the STRING database and visualized using Cytoscape.

All original sequencing data are publicly available in the NCBI Sequence Read Archive under BioProject PRJNA1233427.

### Cell culture

To examine the effects and mechanisms of *P. gingivalis* on peripheral CD4^+^CXCR3^+^ T cells, mouse splenic lymphocytes were divided into three experimental groups: (1) Control group (CON): untreated splenic lymphocytes; (2) *P. gingivalis* group (*P*): splenic lymphocytes stimulated with *P. gingivalis* (1 × 10^8 CFU/mL) for 6 h; and (3) Nuclear factor kappa B **(**NF-κB) inhibitor + *P. gingivalis* group (NF-κB inhibitor + P): splenic lymphocytes pretreated with the NF-κB inhibitor pyrrolidine dithiocarbamate (25 μM; Ambeed, USA) for 30 min, followed by *P. gingivalis* stimulation (1 × 10^8 CFU/mL) for 6 h. This experiment was designed as a stimulus–inhibition assay to determine whether NF-κB inhibition attenuated the response induced by *P. gingivalis* under stimulated conditions. After treatment, CD4^+^ T cells were isolated by negative selection using a CD4^+^T-Cell Isolation Kit (Miltenyi Biotec, Germany) according to the manufacturer's instructions.

### Quantitative reverse-transcription PCR (qRT–PCR)

CD4^+^ T cells were isolated from each group for qRT‒PCR analysis of *Cxcr3*, *Rela* and *Nfkbia* mRNA expression. Total RNA was extracted using RNAiso Plus reagent (Takara Bio, Japan), and RNA concentration and purity were assessed using a spectrophotometer. cDNA was synthesized using the RT Easy™ II Kit (FOREGENE, China), following the manufacturer's instructions. qRT‒PCR was performed on a SLAN-96S Real-Time PCR system (Shanghai Hongshi, China) using a 10 μL reaction mixture containing SYBR qPCR SuperMix Plus (Sichuan Lanyun, China) and gene-specific primers (Chengdu Qingke Biotechnology Co., China).

The amplification protocol included an initial denaturation at 95 °C for 2 min, followed by 40 amplification cycles and a final melt curve analysis. Primer sequences are listed in Table 2. Gene expression was calculated using the 2^-ΔΔCt method.

**Table 2.** Primer sequences for RT–PCR.

Gene		Sequence (5′–3′)
*Cxcr3*	F	TCTGCTGGTGTTAACTCTTC
	R	CCCTGCATAGAAGTTGATGT
*Rela*	F	AGTATTCCTGGCGAGAGA
	R	AACTGTTCCTGGTCCTGT
*Nfkbia*	F	CAGTGTAGCAGTCTTGACG
	R	GATAGAGGCTAGGTGCAGA

### Western blotting

Splenic T cells were lysed using an ultrasonic homogenizer at 30–35 kHz and 50 μA for 2 min with continuous stirring to ensure complete disruption. Total protein concentration was measured using a Bicinchoninic Acid Protein Assay Kit (Solarbio, China). Proteins were precipitated with acetone, dissolved, boiled for 5 min and stored at −70 °C until use.

Next, proteins were separated by molecular weight on a 10% sodium dodecyl sulfate–polyacrylamide gel electrophoresis gel, then transferred onto polyvinylidene fluoride membranes (Merck Millipore, USA) using a Trans-Blot chamber (Bio-Rad, USA). Membranes were blocked with 5% bovine serum albumin in Tris-buffered saline with Tween-20 (TBST) for 1.5 h at 37 °C. Following this, they were incubated for 2 h at 37 °C with primary antibodies diluted in TBST containing 5% bovine serum albumin.

The primary antibody used were as follows: rabbit anti-mouse CXCR3 (1:1000), rabbit anti-mouse NF-κB p65 (1:1000), phospho-NF-κB p65 (1:1000), phospho-IκBα (1:1000), IκBα (1:1000) and tubulin (1:5000) as a loading control.

After washing with TBST, the membranes were incubated with the secondary antibody, biotinylated goat anti-rabbit IgG (Jackson ImmunoResearch, USA), for 1 h at 37 °C. This was followed by treatment with 0.1 µg/mL horseradish peroxidase-conjugated streptavidin (Jackson ImmunoResearch, USA) to enhance the signal. Protein bands were detected using enhanced chemiluminescence reagents (Amersham Healthcare, USA) and visualized with a Vilber Lourmat TFX-35 WL transilluminator. Band intensities were quantified using ImageJ software (NIH, USA) and normalized to tubulin to control for protein loading variability.

### Cell transwell migration experiment

To investigate the effects of *P. gingivalis* on the migratory capacity of peripheral T cells in mice, splenic T cells were divided into three experimental groups: (1) Control group (CON): untreated splenic lymphocytes; (2) *P. gingivalis* group (*P*): splenic lymphocytes stimulated with *P. gingivalis* (1 × 10^8 CFU/mL) for 6 h; and (3) CXCR3 inhibitor + *P. gingivalis* group (CXCR3inhibitor + P): splenic lymphocytes pretreated with the CXCR3 inhibitor AMG 487 (20 μM; final concentration as indicated) for 30 min, followed by *P. gingivalis* (1 × 10^8 CFU/mL) stimulation for 6 h.This experiment was designed as a stimulus–inhibition assay to determine whether CXCR3 inhibition attenuated *P. gingivalis*-induced T-cell migration under stimulated conditions.

After stimulation, CD4⁺ T cells were isolated from the treated splenic lymphocyte suspensions by negative selection (Miltenyi Biotec, Germany) according to the manufacturer's instructions, and the purified T cells were used for the Transwell migration assay. Purified T cells from each group were resuspended in serum-free medium and adjusted to 1 × 10^6^ cells/mL. For the migration assay, the lower chamber of the Transwell insert (5.0 μm pore size; Corning, USA) was filled with medium containing the chemokine CXCL10 (100 ng/mL). A total of 100 μL of the cell suspension was seeded into the upper chamber, and the inserts were incubated at 37 °C with 5% CO₂ for 4 h.

After 4 h, the inserts were removed, the medium was aspirated and the upper surface of the membrane was gently wiped with a PBS-moistened cotton swab to remove non-migrated cells. The inserts were subsequently transferred into a 24-well plate containing 1 mL of 4% paraformaldehyde and fixed for 15 min at room temperature. After discarding the fixative, membranes were washed twice with PBS, stained with 1 mL of crystal violet solution for 10 min and rinsed thrice again with PBS. Migrated cells were then photographed and quantified under a microscope.

### Elisa

The gingipain concentration in gingival tissue homogenates was measured using a commercial Elisa kit (Saipeisen, China), according to the manufacturer's instructions. Briefly, gingival tissues were homogenized in cold PBS and centrifuged to collect supernatants. Samples and standards were added to pre-coated plates and incubated with HRP-conjugated detection antibody. After washing, tetramethylbenzidine (TMB) substrate was applied and the reaction was stopped with stop solution. Absorbance was read at 450 nm using a Thermo microplate reader (Thermo Fisher Scientific, USA).

### Statistical analysis

Statistical analyzes were performed using GraphPad Prism version 9.0 (GraphPad Software, USA). Data are expressed as mean ± standard deviation. Longitudinal neurological scores (Figure 1B) were analyzed using two-way repeated-measures ANOVA with Bonferroni's multiple comparisons test, with time and experimental group as factors. Other comparisons among groups were conducted using one-way analysis of variance followed by Bonferroni's post hoc test. The Bonferroni correction was applied to all pairwise comparisons within each individual dataset. A *p*-value < 0.05 was considered statistically significant. For key outcomes, effect sizes and 95% confidence intervals (CIs) are reported to aid interpretation.

**Figure 1.**
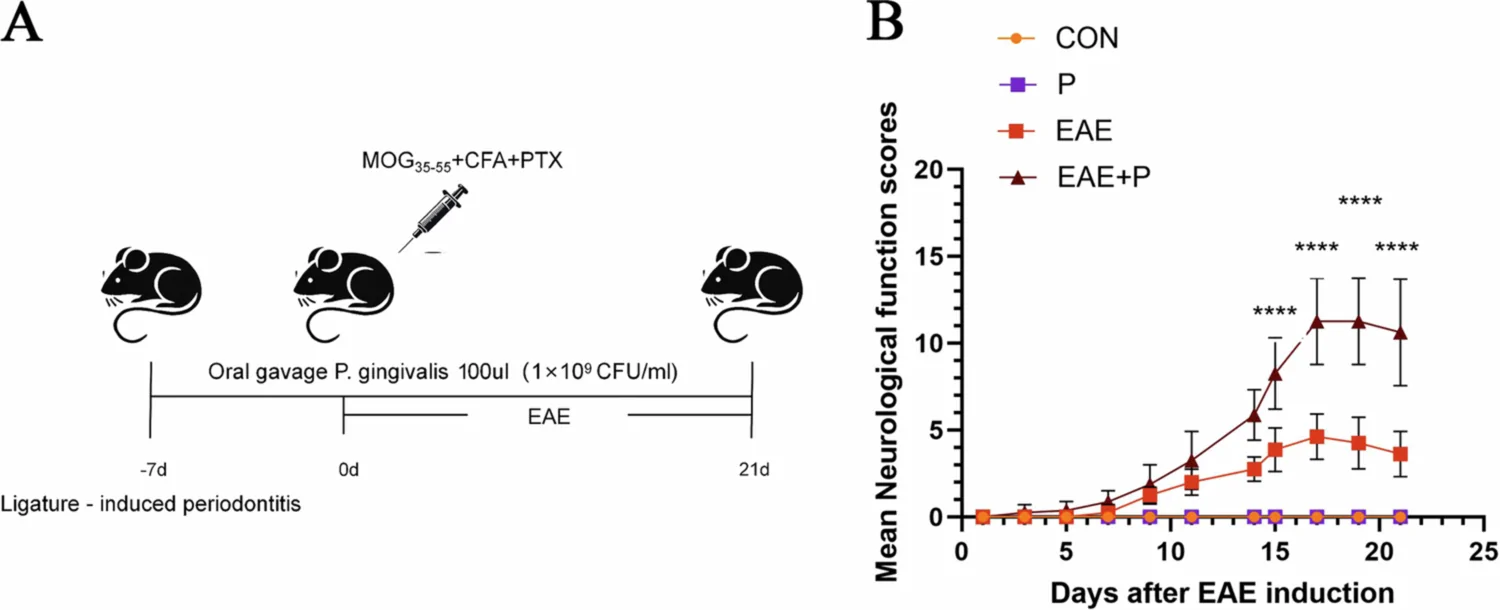
*P. gingivalis-*induced periodontitis significantly exacerbates clinical symptoms of EAE (*n* = 8, *n* = number of mice). (A) Experimental design: On day 0, mice received MOG_35–55_ peptide emulsified in complete Freund's adjuvant and pertussis toxin to induce EAE, followed by monitoring until day 21. Periodontitis was induced by placing 6–0 silk ligatures soaked in *P. gingivalis* around the maxillary second molars 7 days prior to the first EAE induction, followed by daily oral gavage of *P. gingivalis* suspension for 28 consecutive days. (B) Clinical scores from day 0 to day 21. Both the EAE and EAE+P groups developed neurological deficits on day 7, with peak severity on day 17. From day 15 onward, the EAE+P group showed significantly higher neurological scores than the EAE group. Data are presented as mean ± standard deviation (SD). Longitudinal neurological scores were analyzed using two-way repeated-measures ANOVA with Bonferroni's multiple comparisons test. ****p < 0.0001.

## Results

### *P. gingivalis*-induced periodontitis aggravates the clinical signs of EAE in mice

The periodontitis mice model was successfully established by *P. gingivalis* infection and ligation, as confirmed by inflammatory cell infiltration and alveolar bone resorption on H&E staining (Fig. S1). In addition, gingipain levels in gingival tissue homogenates were quantified by Elisa and showed no statistically significant difference between the *P* and EAE+P groups, indicating comparable *P. gingivalis* burden across the two periodontitis conditions (Fig. S2). Weaver's neurological function scale was used to assess clinical signs and confirm successful model establishment in EAE mice. Neurological scores were recorded daily from day 0 to day 21 post-immunization to evaluate the effect of periodontitis on disease progression. Both EAE and EAE+P mice developed neurological deficits by day 7, and peak scores occurred on day 17. Compared with the EAE group, EAE+P mice displayed significantly higher neurological scores from day 15 onward and this difference persisted until day 21 (*p* < 0.0001). This indicates that *P. gingivalis*-induced periodontitis exacerbated clinical symptoms during both the peak and recovery phases of EAE (Figure 1).

### *P. gingivalis*-induced periodontitis increases CD4⁺ T-cell infiltration in the spinal cord of EAE mice

Immunofluorescence staining was used to assess CD4⁺ T-cell infiltration in the spinal cords of mice. At day 21 post-immunization, corresponding to the recovery phase of EAE, the EAE+P group exhibited a significantly higher number of infiltrating CD4⁺ T cells in the spinal cord than the EAE group (*p* < 0.0001). These results indicate that *P. gingivalis*-induced periodontitis promotes the homing of peripheral CD4⁺ T cells to the CNS during EAE recovery (Figure 2).

**Figure 2.**
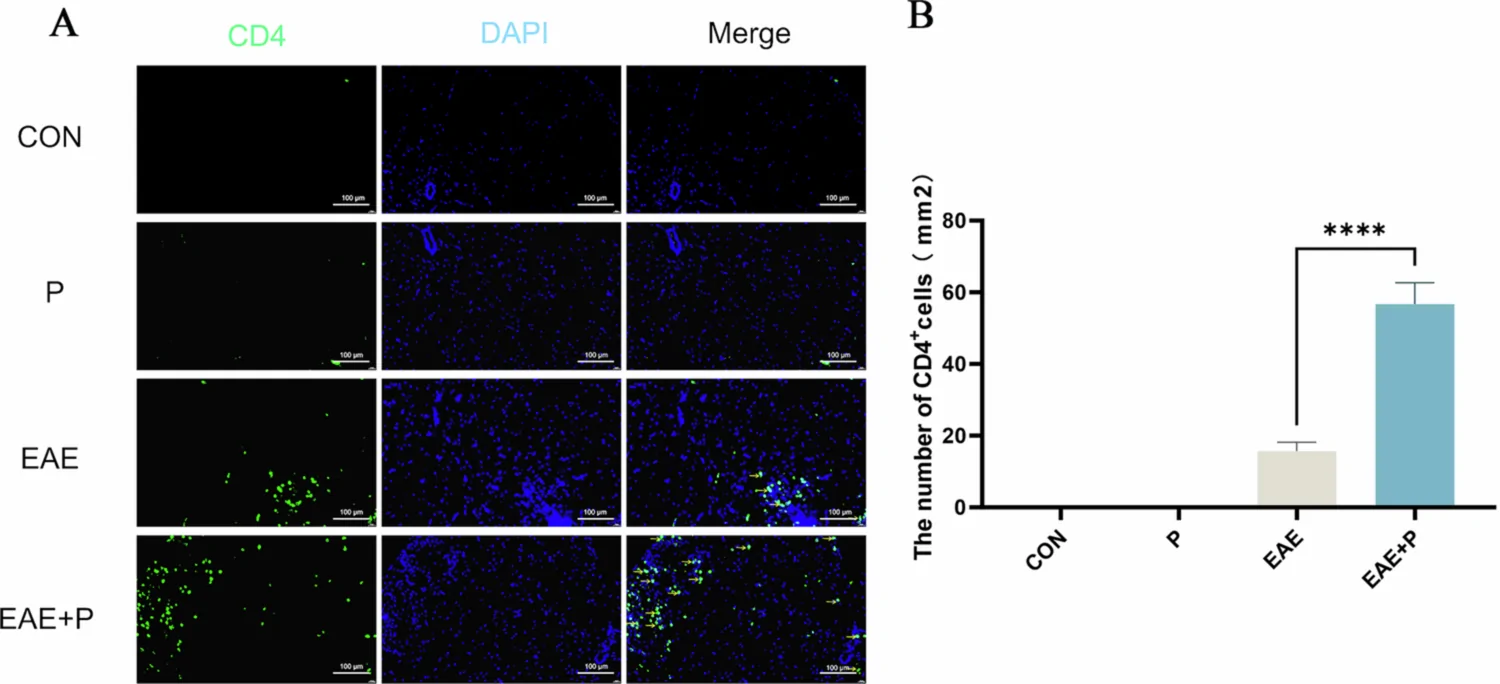
*P. gingivalis*-induced periodontitis exacerbates CD4⁺ T-cell infiltration in the CNS of EAE mice (*n* = 4, *n* = number of mice). (A) Representative immunofluorescence images of spinal cord sections; yellow arrows indicate CD4⁺-positive cells. (B) Quantification of CD4⁺ T cells per mm^2^, presented as mean ± SD. CD4⁺ T-cell infiltration was significantly higher in EAE+P than that in EAE. ****p < 0.0001.

### Transcriptomic data analysis reveals *Cxcr3* upregulation and NF-κB pathway activation in splenic T cells of EAE mice with *P. gingivalis*-induced periodontitis

To investigate how *P. gingivalis*-induced periodontitis modulates systemic T-cell responses in EAE, we reanalyzed transcriptome data from our previously published RNA-seq dataset (NCBI BioProject: PRJNA1233427). This dataset profiled splenic CD4⁺ T cells from EAE and EAE+P mice at the peak of disease (17 dpi).

The reanalysis identified 5,335 DEGs, including 2,297 upregulated and 3,038 downregulated genes in EAE+P relative to EAE. Among these, *Cxcr3* was significantly upregulated in EAE+P, along with several inflammation- and chemokine-related genes such as *Cxcl10*, *Cxcl9*, *Cd4*, *Nfkb1* and *Nfkb2* (*p* < 0.01, *p* < 0.001).

KEGG pathway enrichment revealed that these DEGs were predominantly involved in NF-κB signaling, cell adhesion molecules and T-cell receptor signaling. GO analysis further showed enrichment in biological processes associated with immune regulation, T-cell activation and lymphocyte chemotaxis.

Taken together, these findings suggest that *P. gingivalis*-induced periodontitis enhances Cxcr3 expression in splenic T cells through NF-κB pathway activation. This potentially contributes to the exacerbated systemic immune activation observed in EAE (Figure 3).

**Figure 3.**
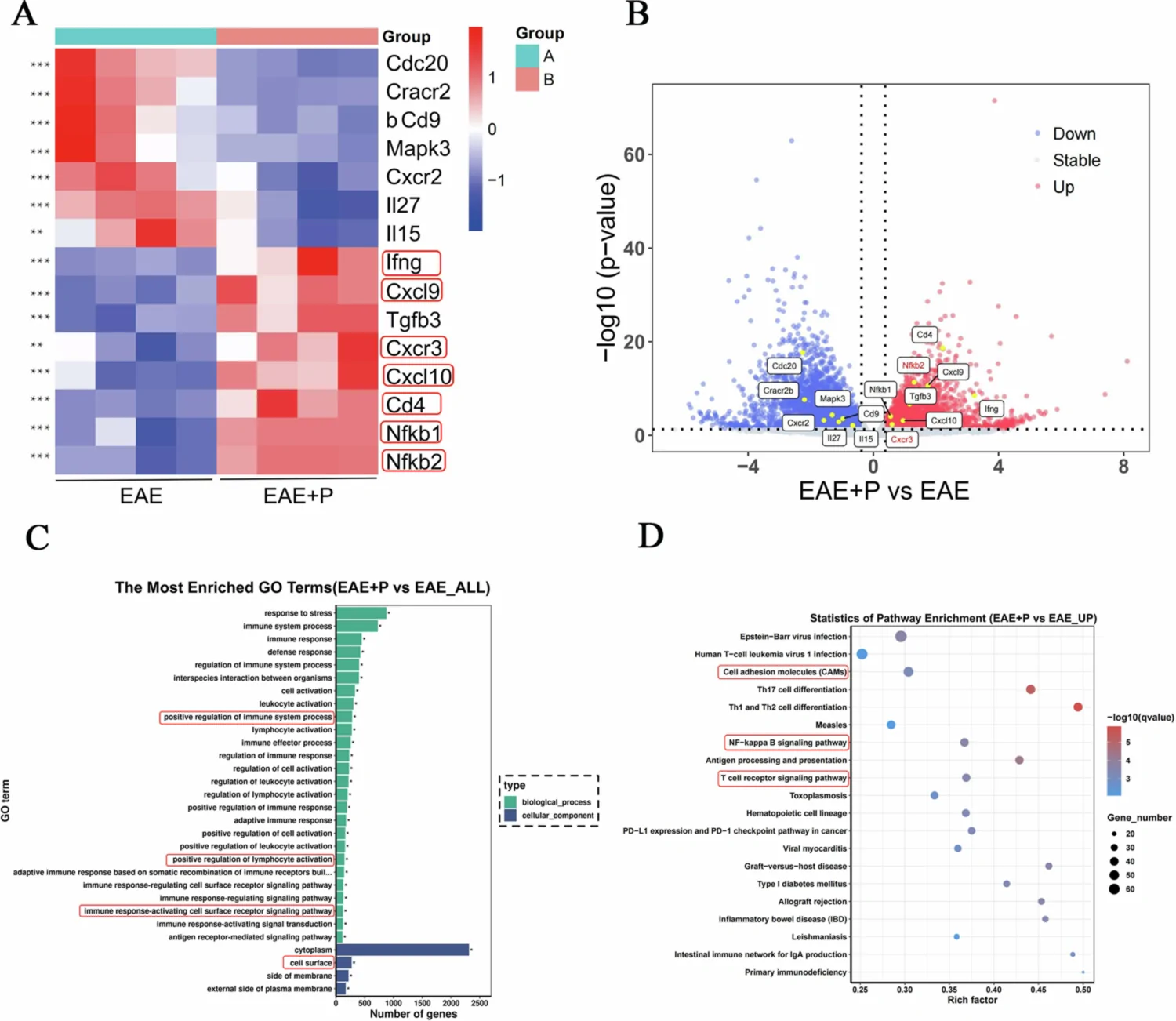
*P. gingivalis*-induced periodontitis enhances *Cxcr3* expression and NF-κB pathway activation in splenic T cells of EAE mice (*n* = 4, *n* = number of mice). (A) Spearman correlation heatmap of differentially expressed genes. Red indicates positive correlation; blue indicates negative correlation. Significantly upregulated genes in EAE+P include *Cxcr3* and *Ifng*, along with those related to chemokine signaling pathways, such as *Cxcl10*, *Cxcl9*, *Cd4*, *Nfkb1* and *Nfkb2*. (B) Volcano plot showing significantly upregulated genes, including *Cxcr3*, *Cxcl10*, *Cxcl9*, *Cd4*, *Nfkb1* and *Nfkb2*. (C, D) KEGG pathway enrichment analysis of differentially expressed genes. EAE+P showed significant enrichment of NF-κB signaling, cell adhesion molecules and T-cell receptor signaling pathways compared with EAE, indicating enhanced activation of these pathways. **p < 0.01, ***p < 0.001.

### *P. gingivalis*-induced periodontitis increased the proportion of CD4^+^CXCR3^+^T cells in the peripheral blood of EAE mice

Given the critical role of CD4⁺CXCR3⁺ T cells in T-cell trafficking during EAE, we assessed their frequency in peripheral blood by flow cytometry to investigate whether *P. gingivalis*-induced periodontitis modulates peripheral immune cell migration. The proportion of CD4⁺CXCR3⁺ T cells was significantly elevated in the *P*, EAE and EAE+P groups compared with the CON group (*p* < 0.01, *p* < 0.001, *p* < 0.0001). Notably, the EAE+P group exhibited a significantly higher frequency of these cells than either the EAE or *P* groups alone (*p* < 0.05, *p* < 0.001). No significant difference was observed between the *P* and EAE groups (Figure 4). These findings indicate that both *P. gingivalis*-induced periodontitis and EAE independently increase the proportion of peripheral CD4⁺CXCR3⁺ T cells, and their combination exerts an additive effect during EAE.

**Figure 4.**
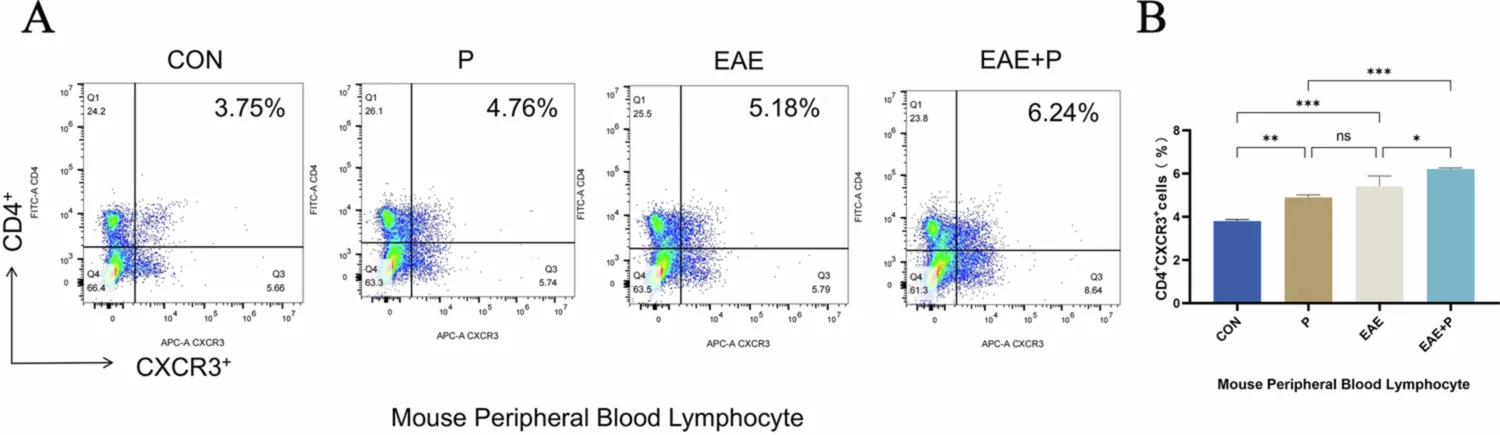
*P. gingivalis*-induced periodontitis increased the proportion of CD4⁺CXCR3⁺ T cells in the peripheral blood of EAE mice (*n* = 4, *n* = number of mice). Frequency of CD4⁺CXCR3⁺ T cells in peripheral blood: (A) Representative dot plots of flow cytometry showing CD4⁺CXCR3⁺ T-cell gating. (B) The frequencies of CD4⁺CXCR3⁺ T cells were significantly increased in the *P*, EAE and EAE+P groups compared with the CON group, and were higher in the EAE+P group than in the EAE or *P* group, while no significant difference was observed between the *P* and EAE groups, presented as mean ± SD. (*p < 0.05, **p < 0.01, ***p < 0.001, NS *p* ≥ 0.05, 95% CI (mean difference) = 0.008–1.282, partial η^2^ = 0.926).

### *P. gingivalis*-induced periodontitis increased the proportion of CD4^+^CXCR3^+^T cells in the splenic lymphocytes of EAE mice

Given the spleen's central role in T-cell activation and chemokine receptor regulation, we next quantified the frequency of CD4⁺CXCR3⁺ T cells within splenic lymphocytes by flow cytometry. The EAE + P group exhibited a significantly higher proportion of CD4⁺CXCR3⁺ T cells compared to the CON, *P* and EAE groups (*p* < 0.0001, *p* < 0.05, *p* < 0.01). Furthermore, both the *P* and EAE groups showed significant increases in this population relative to the CON group (*p* < 0.001), while no statistically significant difference was observed between the *P* and EAE groups (Figure 5). These findings indicate that EAE alone enhances the frequency of splenic CD4⁺CXCR3⁺ T cells, an effect that is further potentiated by concurrent *P. gingivalis*-induced periodontitis.

**Figure 5.**
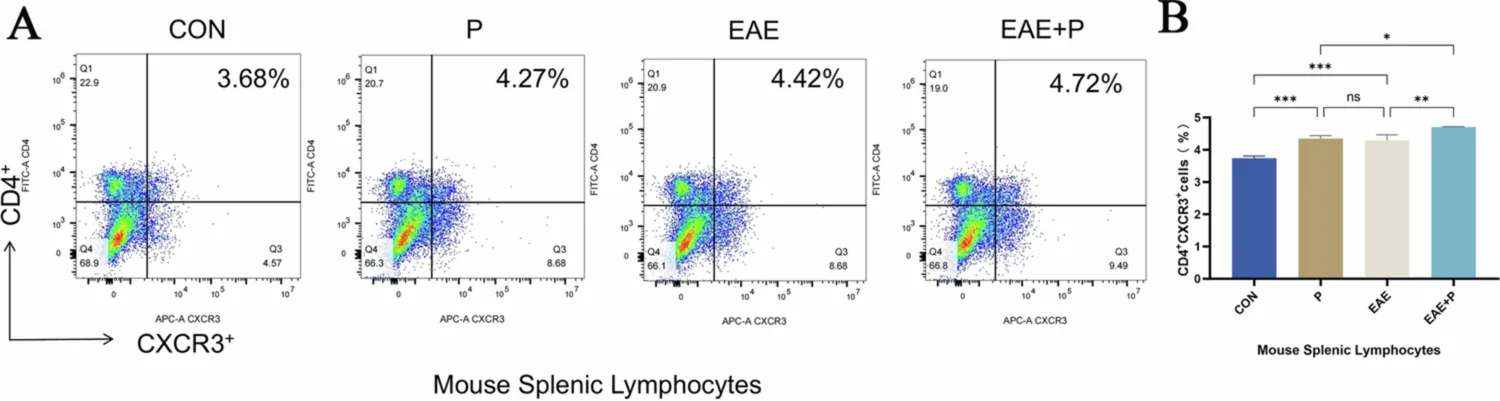
*P. gingivalis*-induced periodontitis increased the proportion of CD4⁺CXCR3⁺ T cells in the splenic lymphocytes of EAE mice (*n* = 4, *n* = number of mice). Frequency of CD4⁺CXCR3⁺ T cells in splenic lymphocytes: (A) Representative dot plots of flow cytometry showing CD4⁺CXCR3⁺ T-cell gating. (B) The EAE+P group exhibited a significantly higher proportion of CD4⁺CXCR3⁺ T cells compared with the CON, *P* and EAE groups, and the EAE group showed a significant increase compared with the CON group, while no significant difference was observed between the *P* and EAE groups, presented as mean ± SD. (*p < 0.05, **p < 0.01, ***p < 0.001, NS *p* ≥ 0.05, 95% CI (mean difference) = 0.134–0.586, partial η^2^ = 0.945).

### *P. gingivalis*-induced periodontitis increases infiltration of CD4^+^CXCR3^+^T cells in the spinal cord of EAE mice

Immunofluorescence staining was performed to assess CD4⁺CXCR3⁺ T-cell infiltration in the spinal cord. At day 21 post-immunization, EAE+P showed a significantly higher number of infiltrating CD4⁺CXCR3⁺ T cells than EAE (*p* < 0.0001) (Figure 6).

**Figure 6.**
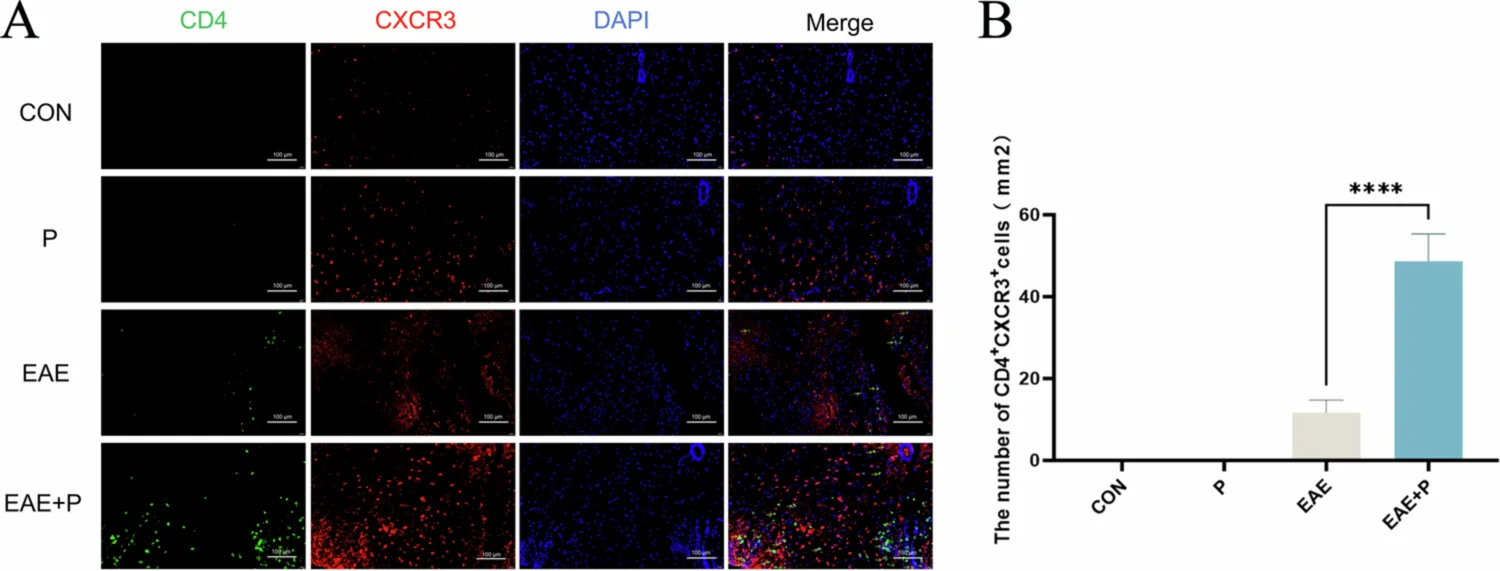
*P. gingivalis*-Induced Periodontitis Increases CD4^+^CXCR3^+^ T-Cell Infiltration in the Spinal Cord of EAE Mice (*n* = 4, *n* = number of mice). (A) Representative immunofluorescence images showing CD4^+^CXCR3^+^ double-positive cells (yellow arrows). (B) Quantification of CD4^+^CXCR3^+^ T cells per mm^2^, presented as mean ± SD. EAE+P displayed a significantly greater number of infiltrating CD4^+^CXCR3^+^ T cells than EAE (****p < 0.0001, 95% CI (mean difference) = 29.66–44.34, partialη^2^ = 0.962).

### *P. gingivalis*-induced periodontitis enhances NF-κB activation (p65 nuclear translocation) in CD4⁺ T cells infiltrating the spinal cord of EAE mice

NF-κB p65 nuclear translocation in spinal cord–infiltrating CD4⁺ T cells was analyzed by immunofluorescence to assess NF-κB activation in CNS-infiltrating T cells in vivo. The proportion of CD4⁺ T cells showing nuclear p65 localization was notably increased in the EAE and EAE+P groups compared to the CON and *P* groups (*p* < 0.0001). Notably, p65 nuclear translocation in spinal cord–infiltrating CD4⁺ T cells was significantly higher in the EAE+P group than in the EAE group (*p* < 0.0001) (Figure 7). These results show that *P. gingivalis*-induced periodontitis is associated with enhanced NF-κB activation (p65 nuclear translocation) in spinal cord–infiltrating CD4⁺ T cells during the disease state of EAE mice.

**Figure 7.**
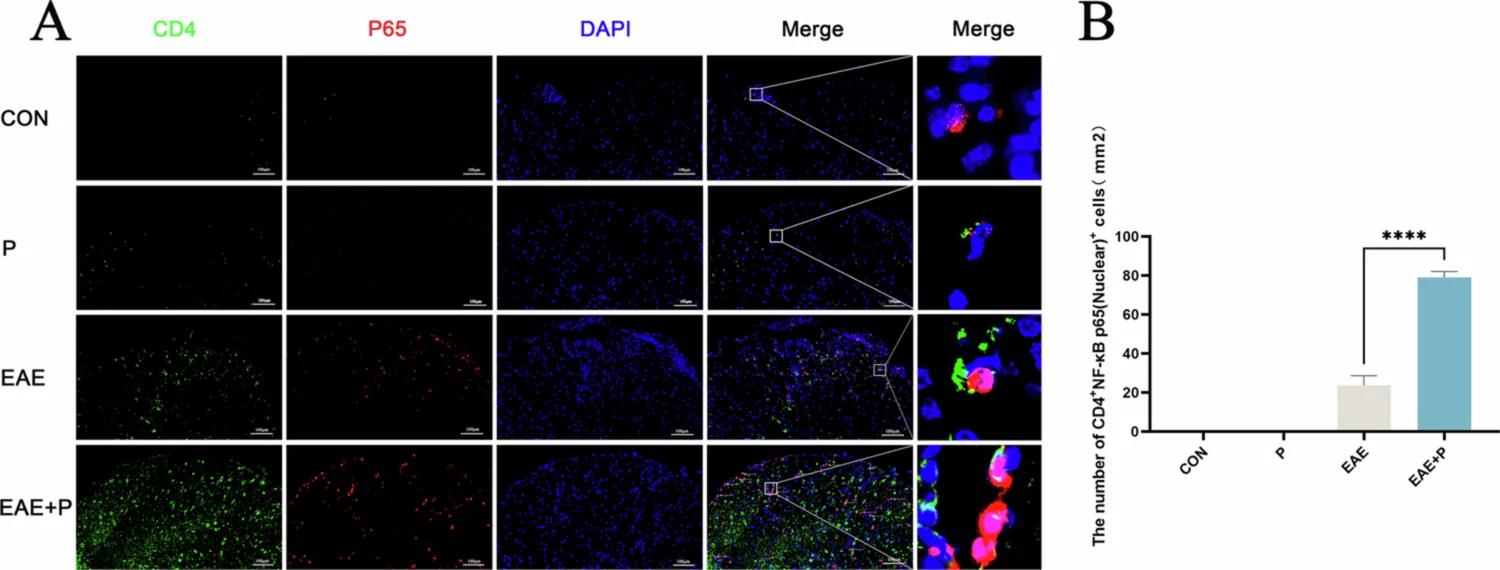
*P. gingivalis*-induced periodontitis enhances NF-κB p65 nuclear translocation in CD4⁺ cells in the spinal cord of EAE mice (*n* = 4, *n* = number of mice). (A) Representative immunofluorescence images showing CD4⁺ cells with NF-κB p65 nuclear translocation (yellow arrows). (B) Quantification of CD4⁺ cells exhibiting NF-κB p65 nuclear translocation per mm^2^, presented as mean ± SD. EAE+P displayed a significantly greater number of CD4⁺ p65 nuclear translocation-positive cells than EAE (****p < 0.0001, 95%CI (mean difference) = 49.72–61.28, partial η^2^ = 0.989).

### *P. gingivalis* enhances the chemotactic ability of peripheral T cells by upregulating CXCR3

To assess whether *P. gingivalis* alters the migratory capacity of splenic T cells, a Transwell migration assay was performed. Compared to the CON group, the *P* group showed a significant increase in the number of migrated T cells following CXCL10 stimulation. In contrast, the CXCR3inhibitor+P group showed markedly reduced T-cell migration compared with the *P* group (*p* < 0.0001) (Figure 8).

**Figure 8.**
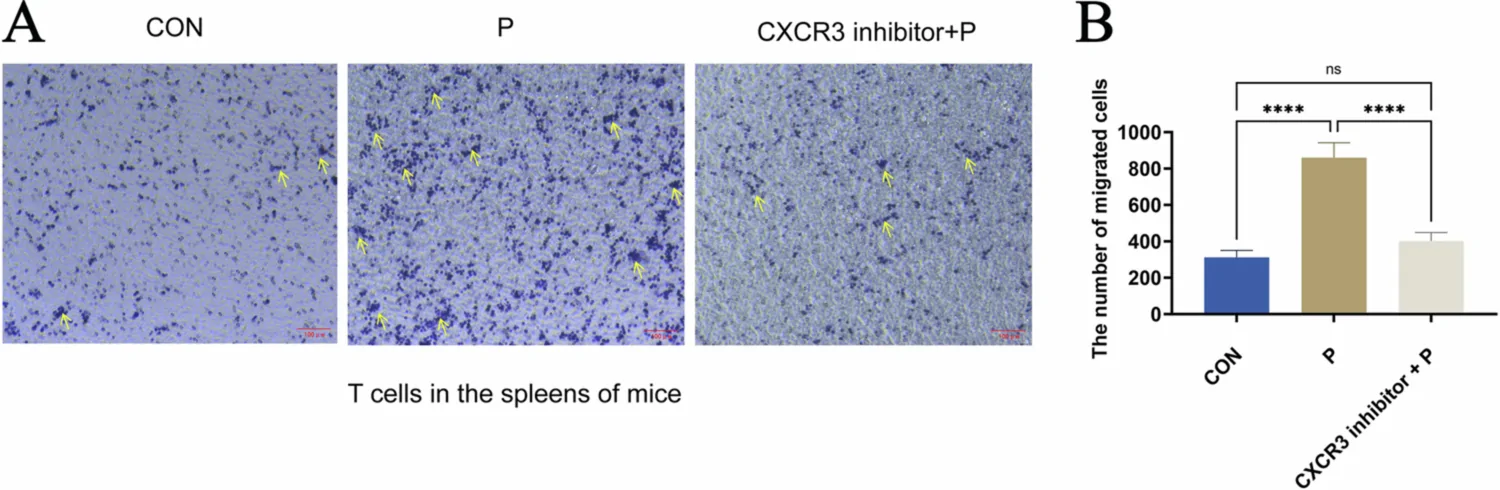
*P. gingivalis* enhances CXCR3-mediated migration of splenic T cells in mice (*n* = 4, *n* = number of mice). (A) Representative microscopic images showing T cells on the lower surface of the Transwell membrane (yellow arrows indicate migrated T cells). (B) Quantification of migrated T cells from five randomly selected fields. Compared with the CON group, the *P* group exhibited a significant increase in the number of migrated T cells. In contrast, the CXCR3inhibitor+P group showed significantly reduced migration compared with the *P* group. The experiments in this figure were performed using cells from the same animals as the corresponding flow cytometry panels in Figures 4 and 5. Data are presented as mean ± SD and were analyzed using one-way analysis of variance (ANOVA). (****p < 0.0001, not significant (NS), *p* ≥ 0.05, 95% CI (mean difference) = 356.91–559.89, partial η^2^ = 0.955).

### *P. gingivalis* increases the proportion of CD4⁺CXCR3⁺T cells in the spleen via an NF-κB pathway

To investigate whether *P. gingivalis* affects the distribution of CXCR3⁺ T-cell subsets in the spleen, we quantified CD4⁺CXCR3⁺T cells in splenic lymphocytes via flow cytometry. The proportion of CD4⁺CXCR3⁺ T cells was significantly increased in the *P* group compared with that in the CON group, indicating that *P. gingivalis* promotes the expansion of CXCR3-expressing CD4⁺ T cells in the spleen.

In the NF-κB inhibitor+P group, the proportion of CD4⁺CXCR3⁺ T cells was significantly reduced compared with that in the *P* group, indicating that the expansion of this subset is at least partially dependent on NF-κB signaling (*p* < 0.0001) (Figure 9).

**Figure 9.**
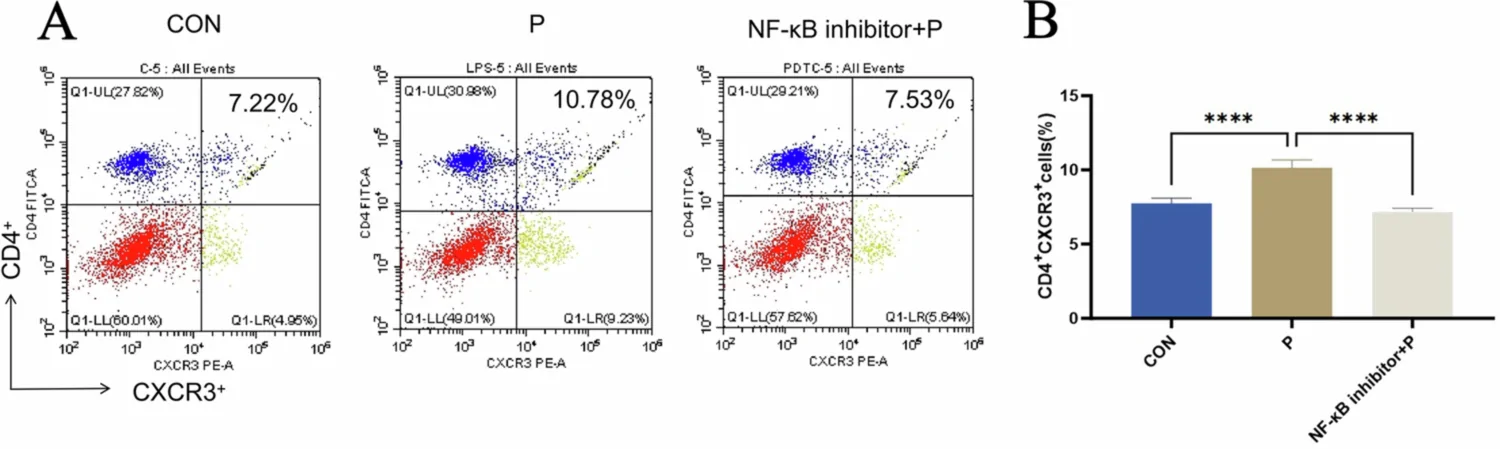
*P. gingivalis* increases the proportion of CD4^+^CXCR3^+^ T cells in splenic lymphocytes of mice (*n* = 4, *n* =  number of mice). (A) Representative dot plots of flow cytometry analysis showing CD4⁺CXCR3⁺ T-cell populations. (B) Quantitative analysis. The *P* group showed a significantly higher proportion of CD4^+^CXCR3^+^T cells than both the CON and NF-κB inhibitor+P groups. The experiments in this figure were performed using cells from the same animals as the corresponding flow cytometry panels in Figures 4 and 5. One-way ANOVA was used for statistical analysis and data are presented as mean ± SD. (****p < 0.0001, 95% CI (mean difference) = 2.313–3.631, partial η^2^ = 0.928).

### *P. gingivalis* activates the NF-κB pathway to enhance CXCR3 expression in T cells

To further elucidate how *P. gingivalis* regulates CXCR3 expression on T-cell surfaces, we conducted *in vitro* T-cell culture experiments. Western blot analysis of mouse splenic T cells revealed significantly increased phosphorylation of p65 and IκBα, along with enhanced IκBα degradation, in the *P* group compared with that in the CON group. Notably, total p65 protein levels remained unchanged across all experimental groups, confirming that the observed increases in p-p65 and nuclear p65 reflect enhanced phosphorylation and nuclear translocation of p65 rather than changes in total p65 expression. These results indicate that *P. gingivalis* stimulation activated the NF-κB signaling pathway.

To verify the critical role of NF-κB signaling in *P. gingivalis*-induced CXCR3 upregulation, an NF-κB inhibitor was added prior to the co-culture of *P. gingivalis* with T cells. Western blot analysis confirmed that inhibition of NF-κB signaling significantly suppressed p65 and IκBα phosphorylation and prevented IκBα degradation, effectively blocking NF-κB activation. Concomitantly, *P. gingivalis*-induced CXCR3 protein and mRNA expression was significantly downregulated, and the proportion of CD4^+^CXCR3^+^T cells was also decreased (*p* < 0.0001) (Figure 10).

**Figure 10.**
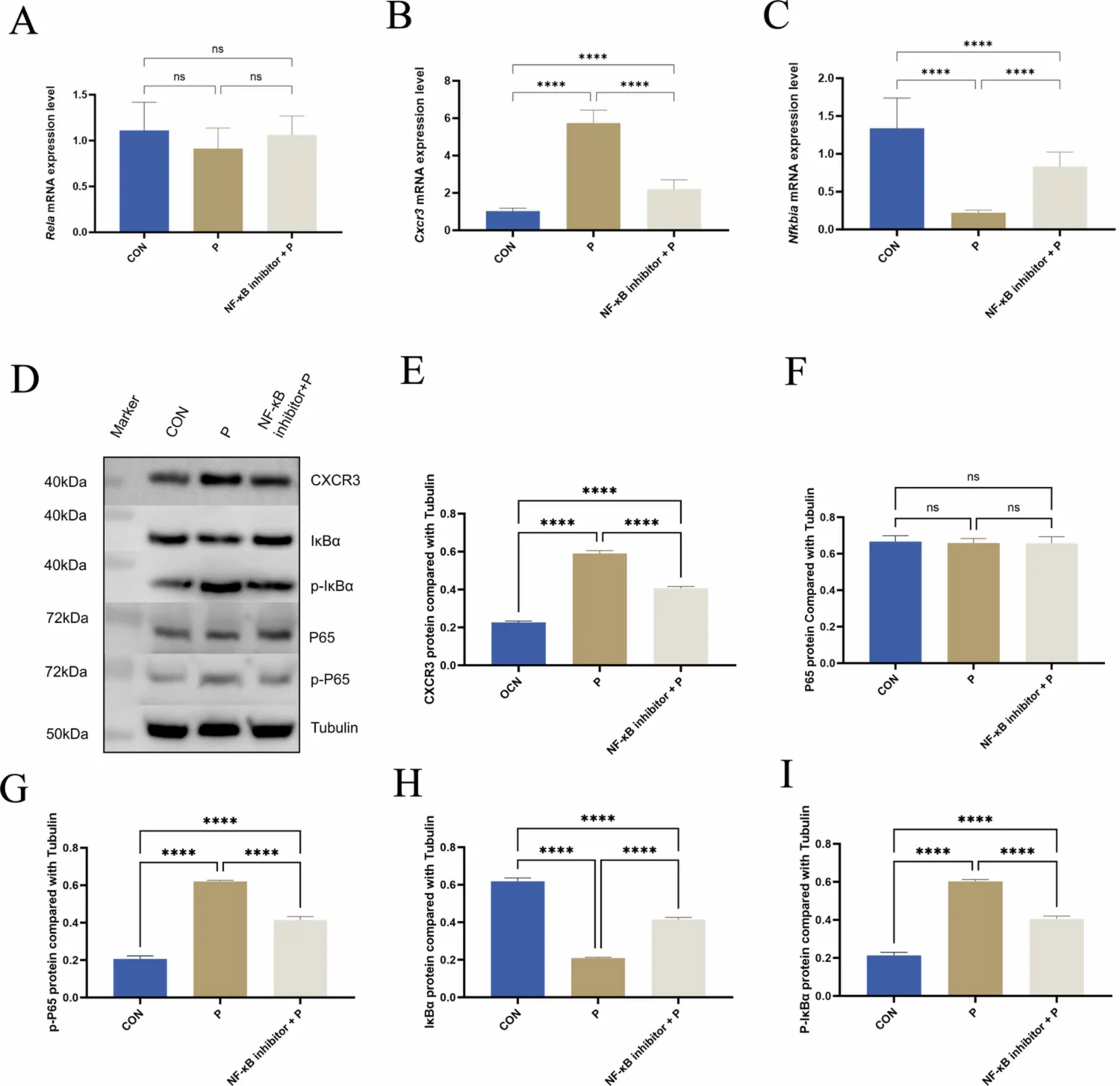
*P. gingivalis* regulates CXCR3 expression on T-cell surface via the NF-κB signaling pathway (*n* = 4, *n* = number of mice). Western blot analysis was used to evaluate the expression levels of CXCR3 and NF-κB pathway-related proteins in splenic T cells. (A-C, E-I) Quantitative analyzes of CXCR3, p65, *p*-p65, IκBα and *p*-IκBα protein and mRNA expression. Compared with the CON and NF-κB inhibitor+P groups, the *P* group exhibited significantly increased expression of CXCR3, *p*-p65 and *p*-IκBα, along with a significant reduction in IκBα expression. (D) Representative western blot images showing CXCR3, p65, phosphorylated NF-κB p65 (*p*-p65), IκBα and phosphorylated IκBα (*p*-IκBα) protein expression. Data were analyzed using one-way ANOVA and are presented as mean ± SD. (****p < 0.0001, NS *p* ≥ 0.05, p-p65:95% CI (mean difference) = 0.1787–0.2333, partial η^2^ = 0.9944).

Altogether, these findings demonstrate that *P. gingivalis* promotes CXCR3 expression on T cells by activating the NF-κB signaling pathway, thereby enhancing the homing capacity of peripheral T cells in EAE mice.

## Discussion

Accumulating evidence suggests that periodontitis is associated with peripheral immune system activation and can influence T-cell compartments [48,49]. Aberrant peripheral T-cell activation and subsequent homing to the central nervous system (CNS) play pivotal roles in the pathogenesis of both multiple sclerosis (MS) and experimental autoimmune encephalomyelitis (EAE). In line with this, we previously demonstrated that *P. gingivalis*-induced periodontitis exacerbates EAE by promoting peripheral Th1 differentiation via ZAP70/NF-κB signaling, accompanied by reduced claudin-5 expression and increased blood–brain barrier (BBB) permeability [48]. Consistent with these findings, our current study shows that concurrent *P. gingivalis*-induced periodontitis significantly worsens the clinical symptoms of EAE during both the peak and recovery phases, as assessed by neurological scoring. Interestingly, the divergence between the EAE and EAE+P groups became more evident during the recovery phase rather than at disease onset. This pattern may suggest that *P. gingivalis*-induced periodontitis does not primarily alter initial EAE induction, but may contribute to sustained peripheral immune activation or delayed resolution of neuroinflammation during later disease stages. Further studies are required to clarify the mechanisms underlying this prolonged inflammatory response. However, the precise mechanisms through which periodontitis modulates peripheral T-cell chemotaxis and CNS homing require further elucidation and more direct evidence.

Extensive evidence indicates that T-cell trafficking across the BBB into the CNS in MS and EAE is a multifactorial process orchestrated by multiple chemokine and adhesion pathways. Within this network, the chemokine receptor CXCR3 and its ligands (CXCL9, CXCL10 and CXCL11) play critical roles by enhancing peripheral T-cell adhesion, facilitating BBB transmigration and promoting CNS infiltration, thereby amplifying local inflammatory responses and neuronal damage [19,39,40,49–51]. CXCR3 represents an important, though not exclusive, homing axis and its relative contribution may vary depending on T-cell subsets, the inflammatory microenvironment and disease stage. Elevated CXCR3 expression has been detected in both the peripheral blood and cerebrospinal fluid of patients with MS, accompanied by increased levels of its ligands (CXCL9/CXCL10/CXCL11) within the CNS [51]. In EAE models, genetic ablation or pharmacological blockade of CXCR3 markedly reduced Th1 cell infiltration into the CNS and alleviated demyelination [49,52], supporting the involvement of CXCR3 in T-cell homing/infiltration to the CNS and in disease progression. Furthermore, CXCR3 is highly expressed in Th1 cells within inflamed tissues, including periodontitis lesions [16]. Notably, systemic administration of CXCR3 antagonists significantly attenuates LPS-induced bone loss, highlighting CXCR3 as a promising therapeutic target [38,53].

In this study, reanalysis of our previously generated transcriptomic data from splenic T cells revealed that *P. gingivalis* infection significantly upregulated *Cxcr3* gene expression in EAE mice. Consistent with these findings, we observed increased proportions of CD4⁺CXCR3⁺ T cells in both peripheral blood and splenic lymphocytes. Collectively, these data confirm that concomitant periodontitis increases CXCR3 expression in peripheral T cells during EAE. Functionally, our in vitro experiments demonstrated that *P. gingivalis* exposure increased the frequency of CXCR3⁺CD4⁺ T cells, elevated CXCR3 protein and mRNA expression and enhanced T-cell chemotaxis, whereas CXCR3 blockade effectively attenuated this migratory response. Overall, these data suggest that *P. gingivalis* enhances CD4⁺ T-cell chemotaxis primarily via CXCR3 upregulation. At the tissue level, we observed increased CD4⁺ T-cell infiltration in the spinal cord of EAE mice with *P. gingivalis*-induced periodontitis, and more specifically, robust infiltration of CD4⁺CXCR3⁺ T cells into the spinal cord was also detected, collectively supporting a mechanistic scheme in which *P. gingivalis*-induced periodontitis drives a CXCR3-skewed peripheral T-cell phenotype with heightened migratory potential and facilitates their CNS homing during EAE.

We next investigated the molecular pathway underlying CXCR3 upregulation in peripheral T cells in EAE mice with *P. gingivalis*-induced periodontitis. Re-analysis of our previously generated transcriptomic sequencing revealed a pronounced elevation of *Cxcr3* expression in peripheral T cells from *P. gingivalis*-infected EAE mice, together with upregulation of NF-κB-associated genes. Enrichment analysis further identified NF-κB signaling as a key pathway activated by *P. gingivalis* infection.

Previous studies have established that NF-κB signaling regulates CXCR3-mediated T-cell migration into the CNS [50], and shared genetic profiles between periodontitis and MS are predominantly enriched in the NF-κB pathway [54,55]. Moreover, *P. gingivalis* LPS is known to activate NF-κB signaling in oral epithelial cells and periodontal ligament stem cells. Consistently, our study demonstrated that *P. gingivalis* stimulation significantly enhanced p65 and IκBα phosphorylation and promoted IκBα degradation in splenic T cells, thereby activating the NF-κB pathway. Correspondingly, we observed increased frequencies of CXCR3⁺CD4⁺ T cells, elevated CXCR3 protein and mRNA expression and enhanced chemotaxis. Importantly, pharmacological inhibition of NF-κB signaling markedly reduced the phosphorylation and degradation processes, resulting in significantly reduced CXCR3⁺CD4⁺ T-cell frequency and CXCR3 expression. These results demonstrate that *P. gingivalis* upregulates CXCR3 expression on T cells through activation of the NF-κB signaling pathway. Consistent with these ex vivo findings, we observed enhanced NF-κB activation in vivo, as evidenced by increased p65 nuclear translocation in CD4⁺ T cells infiltrating the spinal cord of EAE mice with *P. gingivalis*-induced periodontitis.

Taken together, our results support the notion that *P. gingivalis*-induced periodontitis is associated with increased CXCR3 expression on T cells in the context of NF-κB pathway activation, consistent with enhanced T-cell migratory capacity and CNS infiltration and with more severe EAE pathology. This NF-κB–CXCR3 axis may therefore represent a plausible pathway linking peripheral T-cell activation to increased CNS homing potential during EAE, and its upregulation coincides with worse clinical disease severity.

Although we provide supporting in vivo and in vitro evidence, the mechanistic inference is based primarily on in vitro perturbation assays, and inhibitor effects under basal conditions were not independently assessed. In addition, only female mice were studied, limiting the assessment of potential sex-dependent effects. Future studies employing in vivo functional blockade or genetic approaches in both sexes are warranted to strengthen causal inference and generalizability. The specific *P. gingivalis* virulence determinants responsible for the observed effects also warrant further investigation. Despite these limitations, this work provides a useful foundation for subsequent studies exploring the oral–CNS immune axis in autoimmune neuroinflammation.

## Conclusion

In summary, our findings show that *P. gingivalis*-induced periodontitis exacerbates EAE and support a potential NF-κB/CXCR3-mediated mechanism involving NF-κB pathway activation, increased CXCR3 expression on peripheral T cells and enhanced T-cell migratory capacity. Increased infiltration of CD4⁺CXCR3⁺ T cells into the CNS was associated with more severe neurological deficits. These findings suggest that the NF-κB/CXCR3 axis may represent a potential mechanistic link between periodontitis and neuroinflammation, providing new insights into the immunopathological connection between oral infection and MS progression. Targeting this pathway may represent a promising adjunctive therapeutic strategy for MS patients, particularly those with concomitant periodontal disease.

## Supplementary Material

FigS2.tif Supplementary Material

FigS1.tif Supplementary Material

## References

[1] Kunkl M , Amormino C , Tedeschi V , et al. Astrocytes and inflammatory T helper cells: a dangerous liaison in multiple sclerosis. Front Immunol. 2022;13:824411. doi: 10.3389/fimmu.2022.824411 PMC8860818 35211120

[2] Liu R , Du S , Zhao L , et al. Autoreactive lymphocytes in multiple sclerosis: pathogenesis and treatment target. Front Immunol. 2022;13:996469. doi: 10.3389/fimmu.2022.996469 PMC9539795 36211343

[3] McGinley MP , Goldschmidt CH , Rae-Grant AD . Diagnosis and treatment of multiple sclerosis: a review. J Am Med Assoc. 2021;325:765–779. doi: 10.1001/jama.2020.26858 33620411

[4] Walton C , King R , Rechtman L , et al. Rising prevalence of multiple sclerosis worldwide: insights from the Atlas of MS, third edition. Mult Scler. 2020;26:1816–1821. doi: 10.1177/1352458520970841 PMC7720355 33174475

[5] Tian DC , Zhang C , Yuan M , et al. Incidence of multiple sclerosis in China: a nationwide hospital-based study. Lancet Reg Health West Pac. 2020;1:100010. doi: 10.1016/j.lanwpc.2020.100010 PMC8315658 34327341

[6] Zuo M , Ramaglia V , Gommerman JL . Age-related changes in multiple sclerosis and experimental autoimmune encephalomyelitis. Semin Immunol. 2022;59:101631. doi: 10.1016/j.smim.2022.101631 35752572

[7] Bjelobaba I , Begovic-Kupresanin V , Pekovic S , et al. Animal models of multiple sclerosis: focus on experimental autoimmune encephalomyelitis. J Neurosci Res. 2018;96:1021–1042. doi: 10.1002/jnr.24224 29446144

[8] Melamed E , Palmer JL , Fonken C . Advantages and limitations of experimental autoimmune encephalomyelitis in breaking down the role of the gut microbiome in multiple sclerosis. Front Mol Neurosci. 2022;15:1019877. doi: 10.3389/fnmol.2022.1019877 PMC9672668 36407764

[9] Constantinescu CS , Farooqi N , O'Brien K , et al. Experimental autoimmune encephalomyelitis (EAE) as a model for multiple sclerosis (MS). Br J Pharmacol. 2011;164:1079–1106. doi: 10.1111/j.1476-5381.2011.01302.x PMC3229753 21371012

[10] Karpus WJ . Cytokines and chemokines in the pathogenesis of experimental autoimmune encephalomyelitis. J Immunol. 2020;204:316–326. doi: 10.4049/jimmunol.1900914 31907274

[11] Akbar I , Tang R , Baillargeon J , et al. Cutting edge: Serpine1 negatively regulates Th1 cell responses in experimental autoimmune encephalomyelitis. J Immunol. 2023;211:1762–1766. doi: 10.4049/jimmunol.2300526 37909848

[12] Fettig NM , Robinson HG , Allanach JR , et al. Inhibition of Th1 activation and differentiation by dietary guar gum ameliorates experimental autoimmune encephalomyelitis. Cell Rep. 2022;40:111328. doi: 10.1016/j.celrep.2022.111328 36103823

[13] Lv J , Zhuang W , Zhang Y , et al. 9, 10-Anhydrodehydroartemisinin attenuates experimental autoimmune encephalomyelitis by inhibiting Th1 and Th17 cell differentiation. Inflammation. 2021;44:1793–1802. doi: 10.1007/s10753-021-01456-5 33788130

[14] Kunkl M , Frascolla S , Amormino C , et al. T helper cells: the modulators of inflammation in multiple sclerosis. Cells. 2020;9:482. doi: 10.3390/cells9020482 PMC7072830 32093011

[15] Koetzier SC , van Langelaar J , Blok KM , et al. Brain-homing CD4(+) T cells display glucocorticoid-resistant features in MS. Neurol Neuroimmunol Neuroinflamm. 2020;7. doi: 10.1212/nxi.0000000000000894 PMC7577536 33037101

[16] Jenh CH , Cox MA , Cui L , et al. A selective and potent CXCR3 antagonist SCH 546738 attenuates the development of autoimmune diseases and delays graft rejection. BMC Immunol. 2012;13:2. doi: 10.1186/1471-2172-13-2 PMC3298469 22233170

[17] Lalor SJ , Segal BM . Th1-mediated experimental autoimmune encephalomyelitis is CXCR3 independent. Eur J Immunol. 2013;43:2866–2874. doi: 10.1002/eji.201343499 23873018

[18] Steinman L . Capturing pathogenic immune cells before they home to brain. Med. 2021;2:214–216. doi: 10.1016/j.medj.2021.02.005 PMC8011039 33796875

[19] Koper OM , Kamińska J , Sawicki K , et al. CXCL9, CXCL10, CXCL11, and their receptor (CXCR3) in neuroinflammation and neurodegeneration. Adv Clin Exp Med. 2018;27:849–856. doi: 10.17219/acem/68846 29893515

[20] Kwon T , Lamster IB , Levin L . Current concepts in the management of periodontitis. Int Dent J. 2021;71:462–476. doi: 10.1111/idj.12630 PMC9275292 34839889

[21] Dannewitz B , Holtfreter B , Eickholz P . Periodontitis-therapy of a widespread disease. Bundesgesundheitsblatt Gesundheitsforschung Gesundheitsschutz. 2021;64:931–940. 10.1007/s00103-021-03373-2 PMC8264996 34236451

[22] Trindade D , Carvalho R , Machado V , et al. Prevalence of periodontitis in dentate people between 2011 and 2020: a systematic review and meta-analysis of epidemiological studies. J Clin Periodontol. 2023;50:604–626. doi: 10.1111/jcpe.13769 36631982

[23] Chen Y , Lou J , Fang Y , et al. Potential roles and mechanisms of bacterial peptidylarginine deiminase in dental biofilm mediated by *Porphyromonas gingivalis* . J Oral Microbiol. 2025;17:2578893. doi: 10.1080/20002297.2025.2578893 PMC12599369 41221326

[24] Santacroce L , Jirillo E , Bottalico L , et al. Does a link exist between oral microbiota and oral squamous cell carcinoma? A review of current insights. J Oral Microbiol. 2025;17:2569934. doi: 10.1080/20002297.2025.2569934 PMC12599010 41221327

[25] Sanz M , Marco Del Castillo A , Jepsen S , et al. Periodontitis and cardiovascular diseases: consensus report. J Clin Periodontol. 2020;47:268–288. doi: 10.1111/jcpe.13189 PMC7027895 32011025

[26] Baeza M , Morales A , Cisterna C , et al. Effect of periodontal treatment in patients with periodontitis and diabetes: systematic review and meta-analysis. J Appl Oral Sci. 2020;28:e20190248. doi: 10.1590/1678-7757-2019-0248 PMC6919200 31939522

[27] Guo H , Chang S , Pi X , et al. The effect of periodontitis on dementia and cognitive impairment: a meta-analysis. Int J Environ Res Public Health. 2021;18:6823. doi: 10.3390/ijerph18136823 PMC8297088 34202071

[28] Del Pinto R , Landi L , Grassi G , et al. Hypertension and periodontitis: a joint report by the Italian society of hypertension (SIIA) and the Italian society of periodontology and implantology (SIdP). High Blood Press Cardiovasc Prev. 2021;28:427–438. doi: 10.1007/s40292-021-00466-6 PMC8484186 34562228

[29] Nwizu N , Wactawski-Wende J , Genco RJ . Periodontal disease and cancer: epidemiologic studies and possible mechanisms. Periodontol 2000. 2020;83:213–233. doi: 10.1111/prd.12329 PMC7328760 32385885

[30] Ozmeric N , Elgun S , Kalfaoglu D , et al. Interaction between hypertension and periodontitis. Oral Dis. 2024;30:1622–1631. doi: 10.1111/odi.14543 36798972

[31] Tsimpiris A , Tsolianos I , Grigoriadis A , et al. Association of chronic periodontitis with multiple sclerosis: a systematic review and meta-analysis. Mult Scler Relat Disord. 2023;77:104874. doi: 10.1016/j.msard.2023.104874 37478676

[32] Gasmi Benahmed A , Kumar Mujawdiya P , Noor S , et al. *Porphyromonas gingivalis* in the development of periodontitis: impact on dysbiosis and inflammation. Arch Razi Inst. 2022;77:1539–1551. doi: 10.22092/ari.2021.356596.1875 PMC10133641 37123122

[33] Murugaiyan V , Utreja S , Hovey KM , et al. Defining *Porphyromonas gingivalis* strains associated with periodontal disease. Sci Rep. 2024;14:6222. doi: 10.1038/s41598-024-56849-x PMC10940620 38485747

[34] Nuñez-Belmar J , Morales-Olavarria M , Vicencio E , et al. Contribution of -Omics technologies in the study of *Porphyromonas gingivalis* during periodontitis pathogenesis: a minireview. Int J Mol Sci. 2022;24:620. doi: 10.3390/ijms24010620 PMC9820714 36614064

[35] Zangeneh Z , Abdi-Ali A , Khamooshian K , et al. Bacterial variation in the oral microbiota in multiple sclerosis patients. PLoS One. 2021;16:e0260384. doi: 10.1371/journal.pone.0260384 PMC8631616 34847159

[36] Li X , Kiprowska M , Kansara T , et al. Neuroinflammation: a distal consequence of periodontitis. J Dent Res. 2022;101:1441–1449. doi: 10.1177/00220345221102084 PMC9608094 35708472

[37] Polak D , Shmueli A , Brenner T , et al. Oral infection with *P. Gingivalis* exacerbates autoimmune encephalomyelitis. J Periodontol. 2018;89:1461–1466. doi: 10.1002/JPER.17-0531 30030849

[38] Lari S , Hiyari S , de Araújo Silva DN , et al. Local delivery of a CXCR3 antagonist decreases the progression of bone resorption induced by LPS injection in a murine model. Clin Oral Investig. 2022;26:5163–5169. doi: 10.1007/s00784-022-04484-z PMC9710470 35462591

[39] Karin N , Wildbaum G , Thelen M . Biased signaling pathways via CXCR3 control the development and function of CD4+ T cell subsets. J Leukoc Biol. 2016;99:857–862. doi: 10.1189/jlb.2MR0915-441R 26657511

[40] Aloyouny AY , Bepari A , Rahman I . Evaluating the role of CXCR3 in pain modulation: a literature review. J Pain Res. 2020;13:1987–2001. doi: 10.2147/JPR.S254276 PMC7418155 32821152

[41] Mesquita Jr D , Cruvinel WM , Resende LS , et al. Follicular helper T cell in immunity and autoimmunity. Braz J Med Biol Res. 2016;49:e5209. doi: 10.1590/1414-431x20165209 PMC4843212 27096200

[42] Albalawi YA , Narasipura SD , Olivares LJ , et al. CD4(dim) CD8(bright) T cells home to the brain and mediate HIV neuroinvasion. J Virol. 2022;96:e0080422. doi: 10.1128/jvi.00804-22 PMC9364807 35852355

[43] Xiong Y , Ahmad S , Iwami D , et al. T-bet regulates natural regulatory T cell afferent lymphatic migration and suppressive function. J Immunol. 2016;196:2526–2540. doi: 10.4049/jimmunol.1502537 PMC4779695 26880765

[44] Samarakoon A , Shim YA , Dosanjh M , et al. CD45 regulates GM-CSF, retinoic acid and T-cell homing in intestinal inflammation. Mucosal Immunol. 2016;9:1514–1527. doi: 10.1038/mi.2016.23 27007678

[45] Pinho ACO , Laranjeira P , Carvalho E . Role of CD20+ T cells in cancer, autoimmunity and obesity. J Endocrinol. 2024;260:e230242. doi: 10.1530/joe-23-0242 38236766

[46] Lazarević M , Stanisavljević S , Nikolovski N , et al. Complete Freund's adjuvant as a confounding factor in multiple sclerosis research. Front Immunol. 2024;15:1353865. doi: 10.3389/fimmu.2024.1353865 PMC10902151 38426111

[47] Wu X , Zhou M , Ding H , et al. Myelin oligodendrocyte glycoprotein induces aquaporin-4 autoantibodies in mouse experimental autoimmune encephalomyelitis. J Neuroimmunol. 2013;261:1–6. doi: 10.1016/j.jneuroim.2013.03.008 23707078

[48] Dai D , Cao G , Huang S , et al. *Porphyromonas gingivalis* exacerbates experimental autoimmune encephalomyelitis by driving Th1 differentiation via ZAP70/NF-κB signaling. Front Immunol. 2025;16:1549102. doi: 10.3389/fimmu.2025.1549102 PMC11958167 40170858

[49] Kohler RE , Comerford I , Townley S , et al. Antagonism of the chemokine receptors CXCR3 and CXCR4 reduces the pathology of experimental autoimmune encephalomyelitis. Brain Pathol. 2008;18:504–516. doi: 10.1111/j.1750-3639.2008.00154.x PMC8095644 18422759

[50] Sporici R , Issekutz TB . CXCR3 blockade inhibits T-cell migration into the CNS during EAE and prevents development of adoptively transferred, but not actively induced, disease. Eur J Immunol. 2010;40:2751–2761. doi: 10.1002/eji.200939975 21038468

[51] Choileáin SN , Kleinewietfeld M , Raddassi K , et al. CXCR3+ T cells in multiple sclerosis correlate with reduced diversity of the gut microbiome. J Transl Autoimmun. 2020;3:100032. doi: 10.1016/j.jtauto.2019.100032 PMC7388357 32743517

[52] Souza FDS , Freitas NL , Gomes YCP , et al. Following the clues: usefulness of biomarkers of neuroinflammation and neurodegeneration in the investigation of HTLV-1-Associated myelopathy progression. Front Immunol. 2021;12:737941. doi: 10.3389/fimmu.2021.737941 PMC8576432 34764955

[53] Kabashima H , Yoneda M , Nagata K , et al. The presence of chemokine (MCP-1, MIP-1alpha, MIP-1beta, IP-10, RANTES)-positive cells and chemokine receptor (CCR5, CXCR3)-positive cells in inflamed human gingival tissues. Cytokine. 2002;20:70–77. doi: 10.1006/cyto.2002.1985 12445801

[54] Sheu JJ , Lin HC . Association between multiple sclerosis and chronic periodontitis: a population-based pilot study. Eur J Neurol. 2013;20:1053–1059. doi: 10.1111/ene.12103 23398363

[55] Wu E , Cheng M , Zhang X , et al. Exploration of potential shared gene signatures between periodontitis and multiple sclerosis. BMC Oral Health. 2024;24:75. doi: 10.1186/s12903-023-03846-7 PMC10788039 38218802

